# The Relationship Between Anxiety Sensitivity, Emotional States, and Dry Eye Disease Symptom Severity: A Cross-Sectional Study

**DOI:** 10.3390/vision9020036

**Published:** 2025-04-18

**Authors:** Marko Toth, Nataša Jokić-Begić, Sandro Krašić

**Affiliations:** 1 Department of Optometry, University of Applied Sciences Velika Gorica, Zagrebačka 5, 10410 Velika Gorica, Croatia; 2 Faculty of Humanities and Social Sciences, University of Zagreb, Ivana Lučića 3, 10000 Zagreb, Croatia; njbegic@ffzg.hr; 3 Independent Researcher, 10000 Zagreb, Croatia; sandro.krasic.sk@gmail.com

**Keywords:** dry eye disease, DED, anxiety sensitivity, depression, anxiety, stress, ocular surface disease

## Abstract

Dry eye disease (DED) is often comorbid with psychiatric conditions and psychological disturbances like anxiety and depression. The psychological symptoms are mostly considered to be a consequence of DED or a side-effect of medication. However, the possible psychological etiology of DED is seldom explored. This study explores the relationship between anxiety sensitivity (AS), unpleasant emotional states, and the severity of DED symptoms in a healthy general population sample in Croatia. A total of 766 adults (62.27% females) aged between 18 and 88 years completed an online survey consisting of the Ocular Surface Disease Index (OSDI), Anxiety Sensitivity Index (ASI), and Depression Anxiety Stress Scales (DASS21) together with socio-demographic data. The results revealed significant positive correlations between ASI, emotional states, and OSDI (r = 0.25–0.29, *p* < 0.01). Mediation analysis showed that DASS21 significantly mediates the relationship between ASI and OSDI (B = 0.1, CI = [0.004, 0.2]). Highly anxiety sensitive people are more sensitive to DED symptoms, which additionally increases in a state of emotional stress. Thus, DED symptoms are perceived more intensely and frequently than in less sensitive people. Understanding these associations is crucial for comprehensive DED management, indicating potential benefits from addressing psychological health in DED patients and eye health in psychiatric patients.

## 1. Introduction

Dry eye disease (DED) is one of the most widespread ophthalmological and health problems [1,2]. Prevalence of DED in different countries is between 11 and 52%, but global prevalence is 11.6%, or 9.1% when considering only symptoms [3,4,5].

DED is a multifactorial ocular surface disease possibly caused by tear film instability, hyperosmolarity, ocular surface inflammation and damage, or neurosensory abnormalities [6]. DED symptoms are often present without clinical signs [7,8]. Eye dryness symptoms usually include many different sensations like ocular discomfort, visual disturbances, stinging, burning, itching, light sensitivity, and blurred vision [9,10,11]. Patients also report grittiness, excessive tearing or no tearing at all, mucous or crusty sediments, pain, redness, heavy eyelids, inability to cry, discomfort with contact lenses, difficulty reading, working on a computer, and eye fatigue, which is a most common symptom even in negatively diagnosed individuals [12,13,14].

Less noticed psychological and ophthalmological problems often overlap, particularly in DED patients [15,16,17]. Emerging evidence confirms the associations between DED and anxiety and depression problems [18,19,20,21,22,23,24,25,26,27,28,29,30,31,32,33,34,35,36,37,38,39,40,41,42,43]. There are indications that DED symptoms are more associated with psychological and neurological variables than with objective eye conditions [7,23,35,43,44]. Significant associations of DED symptoms and psychological symptoms with no reasonable explanation of the DED symptoms without visible signs of the disease indicate that mental health disorders might be the actual cause of DED [45].

Personality traits associated with neuroticism might play a role in the perception of DED symptoms [46,47,48]. Recently, anxiety sensitivity (AS) is found to be a good predictor of DED symptom severity [49]. This trait presents a tendency to perceive interoceptive body sensations as dangerous or threatening [50]. Highly anxiety sensitive individuals are vigilant and fearful of body symptoms associated with anxiety because of their beliefs that they may cause harmful physical, social, or psychological consequences [51,52]. Despite the common confusion, AS is distinct from trait anxiety, as it particularly concerns the person’s fear of the anxious or panic sensations on the object of fear, based on beliefs about their harmfulness. Trait anxiety, on the other hand, predicts the entire future anxiety based on previous anxious experiences and focuses directly on the object of fear [53,54,55].

AS is well known to be associated with numerous chronic somatic states [49,56,57,58,59,60,61,62,63,64,65,66]. Thus, it is likely that it may also exacerbate the symptoms of DED. Obviously, the DED symptoms are related to different psychological variables associated with anxiety, but their mutual relationship and influence on DED is still not clear, and the studies that consider all these variables are few. This study strives to fill this gap.

This study aims to explore the relation between AS, unpleasant emotional states, and the severity of DED in the normal population. Therefore, the following problems and hypotheses were proposed:

1. Examine the correlation between AS, unpleasant emotional states of depression, anxiety, and stress, and the subjective severity of DED symptoms.

**H_1_:**  *Unpleasant emotional states and DED symptom severity are in a positive statistically significant correlation.*

**H_2_:**  *AS and DED symptom severity are in a positive statistically significant correlation.*

2. Investigate whether the unpleasant emotional states serve as mediators in the relation between AS and the subjective perception of DED symptoms.

**H_3_:**  *Unpleasant emotional states are statistically significant mediators in the relation between anxiety sensitivity and the perceived DED symptoms.*

**H_4_:**  *Among different emotional mediators, stress is the most effective mediator between AS and the severity of DED symptom perception.*

## 2. Materials and Methods

### 2.1. Data Sources/Measurement

The Ocular Surface Disease Index (OSDI, Allergan Inc. Irvine, CA, USA) [67,68], as a measure of DED symptom severity, was set as an outcome. The OSDI is a widely used and validated questionnaire for DED symptoms diagnostics [34,69]. It includes 12 items in three groups: (1) eye dryness symptoms (e.g., eyes that feel gritty); (2) visual function limitations (e.g., problems with reading); and (3) eye discomfort associated with environmental conditions (e.g., eyes felt discomfort in windy conditions). The answers are given on a scale from 0 (none of the time) to 4 (all of the time) for last week. The OSDI score ranges from 0 to 100. An OSDI value larger than 12 indicates at least a mildly dry eye [67,69,70,71]. The validity and reliability of OSDI are generally good or excellent in both usual and online applications [34,72]. The present study showed excellent reliability (α = 0.87).

The Anxiety Sensitivity Index (ASI) [73] is a measure of AS personality trait and served as the predictor. The ASI contains 16 statements (e.g., “It scares me when my heart beats rapidly”) that measure fear from the sensations associated with physiological arousal describing the possible negative consequences of anxiety symptoms. The answers are given on a scale from 0 (very little) to 4 (very much). The total score ranges from 0 to 64. The questionnaire produced excellent psychometric properties in previous research [74] and good reliability (α = 0.89) in the present research.

Depression, Anxiety, and Stress Scales (DASS21) [75] represent a broader measure of psychological distress or, respectively, unpleasant emotional states of depression, anxiety, and stress, which were set as the mediators. Each of the 21 items is answered on a scale from 0 (did not apply to me at all) to 3 (applied to me very much or most of the time). The score can be expressed as a total score, or, respectively, on each scale represented by 7-item scores sum. Examples of items are: “I couldn’t seem to experience any positive feeling at all” (depression), “I felt scared without any good reason” (anxiety), and “I found it difficult to relax” (stress). The reliability is excellent for each scale [76,77], as it is in the present study (α = 0.94).

### 2.2. Procedure

An online cross-sectional study approved by an institutional review board (clearance EPOP-2021-012) was performed on a convenient healthy adult sample. The survey constructed in the SurveyMonkey^®^ (SurveyMonkey Inc. San Mateo, CA, USA) application recruited Croatian-speaking participants via social networks and personal e-mail contacts. Additional participants were recruited by snowball sampling. Data were collected for 30 days. The participation was anonymous and voluntary, and it was possible to quit at any time. The calculated sample size based on the Croatian population size was 385, expecting a proportion of 50% of people with DED symptoms (CI = 95%). This size was achieved during the first week of data collection. However, to ensure enough eligible participants the invitation was repeated two times until a total of 1169 responses were obtained.

The participants were informed about the objective of the study and terms and asked to give their permission to participate. Because of the possible impact on the DED symptoms [35,78,79,80,81], we excluded persons that, in the last 12 months,

1. used psychopharmaceuticals;
2. wore contact lenses;
3. had eye surgery (including laser surgery);
4. had any pathological eye states (e.g., infections, injuries, glaucoma, etc.) that can be directly connected to dry eye symptoms.

### 2.3. Statistical Methods

The data were coded and entered in the database in the Statistical Package for Social Sciences, version 20.0, which was also used for all statistical analyses. Total scores were calculated according to the manuals, respectively, for OSDI, ASI, DASS21, and their scales. The frequencies and percentages were obtained for socio-demographics (Table 1).

The descriptive and normality tests (Kolomogorov–Smirnoff, Shapiro–Wilk) were computed for age, OSDI, ASI, DASS21, and their scales (Table 2).

The mediation model with ASI as a predictor, Depression, Anxiety, and Stress Scales as mediators, and OSDI as an outcome was tested with Model 4 in the mediation analysis PROCESS macro [82].

Participants with significant data missing in the predictor, mediator, or outcome variables (e.g., the whole OSDI was not answered) were excluded listwise. However, for the cases in which only a few answers were missing, and at the same time other answers were more than zero, missing answers were treated as zero (e.g., only one missing answer in DASS21 was treated as 0 = “Did not apply to me at all”).

## 3. Results

### 3.1. Participants

**Figure 1:**
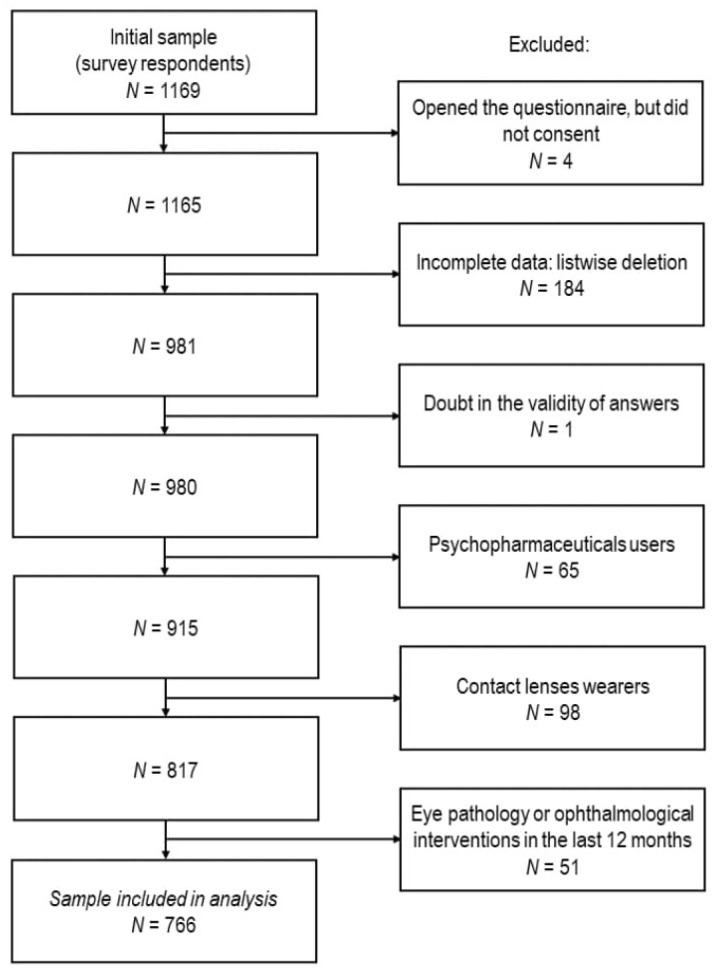
Participant flow chart (N = number of participants) in a cross-sectional online survey.

A total of 1169 people responded to the study. However, 4 did not give their consent, 184 did not complete their surveys, and 1 was excluded because of the doubt about the validity of the answers. Additionally, 65 were using psychopharmaceuticals, 98 wore contact lenses, and 51 had an eye pathology/surgery, and thus were not eligible for the study. In the end the total of 766 people were included in the statistical analysis (Figure 1).

The sample included people between 18 and 88 years (M = 36.25, SD  =  12.99), 62.27% females and 37.4% males. Most of them were between 25 and 54 years old (64.1%) and highly educated (61.7%). Demographics are presented in Table 1.

### 3.2. Descriptive Data

The descriptives and normality distribution tests are presented in Table 2.

The outcome, OSDI, was 19.5 (SD = 14.62) on average and nearly identical to the previous results in a similar Croatian sample [52] and a small Chinese sample [83], but higher than the results on other, non-clinical samples [84,85] and lower compared to the most clinical samples [72,86,87,88,89]. A total of 63.97% of the sample had at least mild dry eye symptoms. A total of 19.84% of participants reported severe symptoms (Table 3).

The average result on predictor ASI was 17.43 (SD = 10.27) and slightly lower than the results on similar Croatian and other samples [49,50,55,74,90,91,92]. This may be due to the larger percentage of women in the sample, who generally score higher on ASI [55,93].

Depression, anxiety, and stress, as mediators, were on average 3.47 (SD = 3.8), 2.64 (SD = 3.24), and 5.18 (SD = 4.25), respectively. Similar results were obtained in a Croatian [49] and a British sample [94], and lower in an Australian normative sample [95].

### 3.3. Intercorrelations and Mediation Analysis

Table 4 shows the intercorrelations of measured variables. The OSDI is weakly, but significantly correlated with all psychological measures (r = 0.25 to 0.29, *p* < 0.01), and most strongly with stress (r = 0.29, *p* < 0.01). AS is in a moderate correlation with unpleasant emotional states.

**Figure 2:**
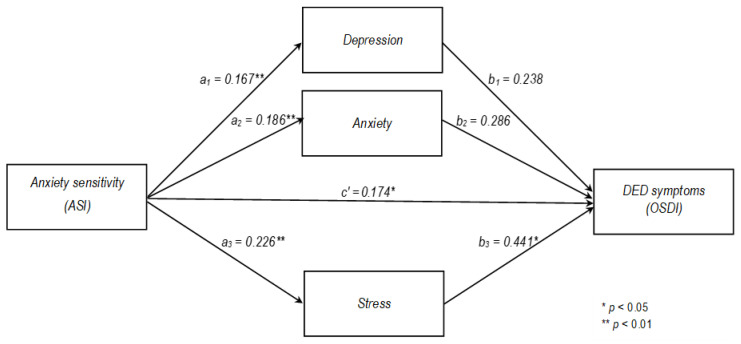
Estimated mediation model of anxiety sensitivity (ASI) on the perception of severity of Dry Eye Disease (DED) symptoms (OSDI) with unpleasant emotional states (depression, anxiety, and stress) as mediators in a cross-sectional study (N = 766).

The mediation analysis with AS as a predictor, and depression, anxiety, and stress emotional states as separate mediators explained 7% (R2 = 0.066 *p* < 0.01) of the DED symptom (OSDI) variance. The results showed a significant total indirect effect of AS on DED symptoms perception via unpleasant emotions (B = 0.192, [0.111, 0.279]), but only indirect effect via emotional state of stress (B = 0.1, CI = [0.004, 0.2]), while the other two mediators were not significant. AS also achieved a direct effect on the perception of DED symptom severity (B = 0.174, CI = [0.052, 0.296]). In conclusion, the results discovered a partial mediation model in which stress has a mediation effect on the association between AS and DED symptoms. The model is presented in Figure 2 and the mediation analysis results are in Table 5.

## 4. Discussion

The results revealed significant but weak correlations between psychological variables and the severity of DED symptoms and confirmed the first two hypotheses. The more intensely unpleasant the emotional states (depression, anxiety, and stress), the more severe DED symptoms are expressed. Furthermore, individuals more sensitive to the symptoms of anxiety and interoceptive sensations are also more sensitive to DED symptoms and perceive them more severely and frequently.

Similar, but higher correlations between OSDI and DASS21 scales were obtained on younger ophthalmology patients [18]. Other, differently designed research implies similar results. Some revealed higher depression, anxiety, and stress in DED patients than in controls [41], and others significant correlation between OSDI and depression [23,96,97]. The correlations of OSDI with anxiety are mostly smaller but still significant [96,97]. In our research, among the DASS21 scales, the largest correlation was between stress and OSDI. This information is rarely available and reported in the literature, but one research revealed strikingly similar results [49]. Thus, stress may be most associated with DED symptoms, but under-investigated.

Some earlier research confirmed significant correlations between AS and dry eyes [49,98]. A significant correlation between non-ocular pain and OSDI was discovered, and it was proposed that certain persons suffer from central sensibilization or sensitivity which affects the pain experience [23]. We can only assume that this central sensitivity is nothing more than anxiety or interoceptive sensitivity, but further evidence is necessary. Our results are the latest addition to the emerging body of evidence that implies the association of DED and psychological symptoms typical for depression and anxiety [7,17,18,19,21,23,24,25,26,27,28,29,30,32,33,35,37,39,41,45,47,78,98,99,100,101,102,103,104,105,106]. Even though these problems have been researched for over two decades now, much is still unknown. Our results make valuable contributions to this field: many DED symptoms can be attributed to constitutional sensitivity.

Recent research detected more DED symptoms in controls than in the group with newly diagnosed depression and comorbid anxiety, which had more clinical signs of DED [36]. This might be an indication that DED symptoms in healthy populations are mostly determined by personality traits (e.g., AS), while psychological disorders affect eyes more directly by a neurophysiological disbalance. A possible explanation is that psychological stress accompanying depression and anxiety can suppress lacrimal gland function and reduce tear production [33].

Our results regarding the second problem follow the same lead. The AS affects the DED symptoms through unpleasant emotional states, and among them through psychological stress the most. Therefore, our third and fourth hypothesis is confirmed. AS also has a direct effect on the perception of DED symptoms severity; thus, the partial complementary mediation model was discovered, which is a valuable addition to our results. These results imply that people who are more sensitive to anxiety symptoms are also sensitive to ocular symptoms and inclined to be on alert for those symptoms, among other body sensations. DED symptoms are also perceived more intensely than in less sensitive people. At the same time, due to emotional stress, the sensitivity to ocular symptoms increases and they are perceived even more intensively.

The results are, at some level, comparable with the research that showed that neurotic personality traits affect DED symptoms [47,48]. AS, emotional states, and DED symptoms are in a similar relation as neurotic personality, emotional states, and DED symptoms [107]. It is also similarly associated with chronic physical states involving unpleasant and painful symptoms [56,57,58,59,60,61,62,63,64,65,66,108,109] and DED patients are like patients with chronic pain somatic syndromes [43]. AS increases pain sensitivity [110,111,112] in the same manner that it increases the sensitivity to DED symptoms, including ocular pain. Also, unpleasant emotional states play a role in pain perception [113,114] which is a frequent symptom of DED. This research indicates the same pattern in accordance with our results and the theory of AS [50,51]. Our research proves the role of AS in ocular problems directly and through its effect on the experience of psychosocial stress. This suggests that AS might be one of the possible explanations of a symptomatic dry eye, but also that DED symptoms are a possible manifestation of AS.

The data collection process in this research allowed for a random selection of the participants, especially younger ones. The additional participants of older age were recruited via personal e-mail contacts and asked to forward the questionnaire to similar groups. Despite the invested effort, this study is not without limitations or completely bias-free. The self-selection of the participants resulted in a larger percentage of younger people and women, meaning that these results should be generalized with caution.

As an online survey, this study includes the self-selection of mostly well-educated, younger, and female participants, which narrows the generalization of the results. The participants were carefully recruited from a healthy population, but all DED risk factors (e.g., computer usage or physical activity) that could affect the results were not excluded [115,116,117,118]. Female gender is a well-known DED risk factor, but a larger percentage of women in this sample is in a certain sense representative of the research purpose. Although online surveys have some economic advantages, self-selection bias affects estimates of psychopathology, such as symptom level or prevalence, even though the effect on associations with putative risk factors is small [119].

Rigorously methodologically speaking, a cross-sectional and correlational study should not allow causal conclusions. Traditionally, there is the demand that predictors temporally precede the outcome [120]. However, the approach based on bootstrapping has significant advantages compared to traditional mediation analysis, and a good theoretical background or a firm argument allows causal conclusions [82,121]. In that sense, we are firm that the predictor AS, as a conative personality trait, and emotional states as mediators are theoretically and logically valid antecedents of DED symptoms. Nevertheless, completely reliable conclusions would be possible only in longitudinal research.

## 5. Conclusions

This study is among the first studies on a large and healthy Croatian sample exploring the symptoms of DED and its relations with psychological variables. However, according to similar findings in other research, these results may also apply to other populations. The study detected significant correlations between psychological characteristics and the severity of DED symptoms and their mutual relation in a large healthy sample. The AS has a direct effect on the perception of DED symptoms severity but also achieves its effect on the symptoms through unpleasant emotional states and emotional stress. The results confirmed that psychological stress can serve as a mediator between personality traits that indicate sensitivity to body symptoms (AS) and the perception of DED severity. People with high anxiety sensitivity are more sensitive to ocular symptoms, among other body symptoms. They are on alert to detect and perceive those symptoms more frequently and intensely than less sensitive people. Additionally, while experiencing emotional stress, the sensitivity to ocular symptoms increases and they are perceived even more severely. Since ocular symptoms may indicate psychological problems and vice versa, the results have diagnostical implications. By applying screening questionnaires in eye and mental care the patients could be pointed toward the appropriate treatment. This may also impact the symptomatic DED treatment because these results suggest that focus on psychological etiology may bring better results. Due to the lack of definitive explanations of associations of psychological and ocular symptoms, this and similar research efforts make an appeal in favor of a much-needed interdisciplinary approach and the collaboration of eye and mental care experts.

## Figures and Tables

**Table 1:** The sample characteristics (N = 766) in the cross-sectional online survey.

Characteristic	Frequency	Percentage
Age:		
18–24	177	23.11
25–34	226	29.5
35–44	165	21.54
45–54	100	13.06
55–64	75	9.79
65–74	15	1.96
75–84	2	0.26
>85	1	0.13
Not answered	5	0.65
Gender:		
Female	477	62.27
Male	286	37.34
Prefer not to say	3	0.39
Education:		
High school	292	38.1
BA or MA	387	50.5
PhD	86	11.2
Not answered	1	0.1

**Table 2:** The descriptive data and distribution normality tests for age, outcome Ocular Surface Disease Index (OSDI), predictor Anxiety Sensitivity Index (ASI), and Depression, Anxiety, and Stress Scales, (DASS21, Dep, Anx, Stress) as mediators in a cross-sectional online study (N = 766).

Variable	M	SD	KS	SW
Age	36.25	12.99	0.103 **	0.932 **
OSDI	19.5	14.62	0.107 **	0.937 **
ASI	17.43	10.27	0.072 **	0.966 **
DASS21	11.28	10.13	0.147 **	0.869 **
Dep	3.47	3.8	0.191 **	0.819 **
Anx	2.64	3.24	0.208 **	0.778 **
Stress	5.18	4.25	0.111 **	0.916 **

M = mean; SD = Standard Deviation; ** *p* < 0.01; K-S = Kolmogorov–Smirnov normality test; S-W = Shapiro–Wilk normality test; OSDI = Ocular Surface Disease Index; ASI = Anxiety Sensitivity Index; DASS21 = Depression, Anxiety and Stress Scales, short version; Dep = DASS21 depression scale; Anx = DASS21 anxiety scale; Stress = DASS21 stress scale.

**Table 3:** Dry Eye Disease (DED) symptom severity distribution according to the OSDI categorization in a cross-sectional online study (N = 766).

DED Category	Cut-Offs	Frequency	Percentage
Normal	≤12	276	36.03
Mild	13–22	191	24.94
Moderate	23–32	147	19.19
Severe	≥33	152	19.84

**Table 4:** Intercorrelations of psychological variables (ASI, depression, anxiety, and stress) with the DED symptom severity perception (OSDI) in a cross-sectional online study (N = 766).

Variable		1	2	3	4
1	ASI	–			
2	Dep	0.45 **	–		
3	Anx	0.59 **	0.65 **	–	
4	Stress	0.55 **	0.74 **	0.73 **	–
5	OSDI	0.26 **	0.25 **	0.27 **	0.29 **

** = *p* < 0.01; ASI = Anxiety Sensitivity Index; OSDI = Ocular Surface Disease Index; Dep = depression; Anx = anxiety; Stress = stress (Depression, Anxiety and Stress Scales, short version).

**Table 5:** Mediation analysis results of the model with ASI as predictor, OSDI as outcome, and depression, anxiety, and stress as mediators (N = 766).

Effect	B	CI	t
Indirect (depression = a_1_ × b_1_)	0.04	[−0.032, 0.115]	
Indirect (anxiety = a_2_ × b_2_)	0.053	[−0.049, 0.159]	
Indirect (stress = a_3_ × b_3_)	0.1 *	[0.004, 0.2]	
Total indirect effect (depression + anxiety + stress)	0.192 *	[0.111, 0.279]	
Direct (c′)	0.174 *	[0.052, 0.296]	2.807 **
Total (depression + anxiety + stress + c′)	0.367 *	[0.269, 0.464]	7.372 **

B = unstandardized coefficients; CI = 95% bootstrap confidence interval; t—t-statistic, * = significance of CI *p* < 0.05; ** significance of t-statistic *p* < 0.01.

## Data Availability

The original contributions presented in this study are included in this article. Further inquiries can be directed to the corresponding authors.

## Abbreviations

The following abbreviations are used in this manuscript:

DED	Dry Eye Disease
AS	Anxiety Sensitivity
ASI	Anxiety Sensitivity Index
OSDI	Ocular Surface Disease Index
DASS21	Depression, Anxiety, and Stress Scales, short version
